# Efficacy of artemether-lumefantrine and dihydroartemisinin-piperaquine and prevalence of molecular markers of anti-malarial drug resistance in children in Togo in 2021

**DOI:** 10.1186/s12936-024-04922-1

**Published:** 2024-04-03

**Authors:** Ameyo Monique Dorkenoo, Marian Warsame, Essoham Ataba, Manani Hemou, Kossi Yakpa, Efoe Sossou, M’badi Mitigmsagou, Carmel Diwaba Teou, Emmanuelle Caspar, Laurence Ma, Koffi Edem Djadou, Tinah Atcha-Oubou, Charlotte Rasmussen, Didier Menard

**Affiliations:** 1 Faculté des Sciences de la Santé, Université de Lomé, Lomé, Togo; 2 School of Public Health and Community Medicine, University of Gothenburg, Gothenburg, Sweden; 3 Programme National de Lutte Contre le Paludisme, Lomé, Togo; 4 Service de Pédiatrie, Centre Hospitalier Universitaire Campus, Lomé, Togo; 5 Service des Laboratoires, Centre Hospitalier Universitaire Sylvanus Olympio Lomé, Lomé, Togo; 6 Institute of Parasitology and Tropical Diseases, Université de Strasbourg, UR7292 Dynamics of Host-Pathogen Interactions, 67000 Strasbourg, France; 7 Biomics Platform, C2RT, Institut Pasteur, 75015 Paris, France; 8 Global Malaria Programme, World Health Organization, Geneva, Switzerland; 9 Malaria Genetics and Resistance Unit, Institut Pasteur, Université Paris Cité, INSERM U1201, 75015 Paris, France; 10 Malaria Parasite Biology and Vaccines, Institut Pasteur, Université Paris Cité, 75015 Paris, France; 11 Laboratory of Parasitology and Medical Mycology, CHU Strasbourg, 67000 Strasbourg, France

**Keywords:** Artemether-lumefantrine, Dihydroartemisinin-piperaquine, *Plasmodium falciparum*, Efficacy, Molecular markers of antimalarial drug resistance, Togo

## Abstract

**Background:**

Artemether-lumefantrine (AL) and dihydroartemisinin-piperaquine (DP) are the currently recommended first- and second-line therapies for uncomplicated *Plasmodium falciparum* infections in Togo. This study assessed the efficacy of these combinations, the proportion of Day3-positive patients (D3 +), the proportion of molecular markers associated with *P. falciparum* resistance to anti-malarial drugs, and the variable performance of HRP2-based malaria rapid diagnostic tests (RDTs).

**Methods:**

A single arm prospective study evaluating the efficacy of AL and DP was conducted at two sites (Kouvé and Anié) from September 2021 to January 2022. Eligible children were enrolled, randomly assigned to treatment at each site and followed up for 42 days after treatment initiation. The primary endpoint was polymerase chain reaction (PCR) adjusted adequate clinical and parasitological response (ACPR). At day 0, samples were analysed for mutations in the *Pfkelch13*, *Pfcrt*, *Pfmdr-1*, *dhfr*, *dhps*, and deletions in the *hrp2/hrp3* genes.

**Results:**

A total of 179 and 178 children were included in the AL and DP groups, respectively. After PCR correction, cure rates of patients treated with AL were 97.5% (91.4–99.7) at day 28 in Kouvé and 98.6% (92.4–100) in Anié, whereas 96.4% (CI 95%: 89.1–98.8) and 97.3% (CI 95%: 89.5–99.3) were observed at day 42 in Kouvé and Anié, respectively. The cure rates of patients treated with DP at day 42 were 98.9% (CI 95%: 92.1–99.8) in Kouvé and 100% in Anié. The proportion of patients with parasites on day 3 (D3 +) was 8.5% in AL and 2.6% in DP groups in Anié and 4.3% in AL and 2.1% DP groups in Kouvé. Of the 357 day 0 samples, 99.2% carried the *Pfkelch13* wild-type allele. Two isolates carried nonsynonymous mutations not known to be associated with artemisinin partial resistance (ART-R) (A578S and A557S). Most samples carried the *Pfcrt* wild-type allele (97.2%). The most common *Pfmdr-1* allele was the single mutant 184F (75.6%). Among *dhfr/dhps* mutations, the quintuple mutant haplotype N51I/C59R/S108N + 437G/540E, which is responsible for SP treatment failure in adults and children, was not detected. Single deletions in *hrp2* and *hrp3* genes were detected in 1/357 (0.3%) and 1/357 (0.3%), respectively. Dual *hrp2/hrp3* deletions, which could affect the performances of HRP2-based RDTs, were not observed.

**Conclusion:**

The results of this study confirm that the AL and DP treatments are highly effective. The absence of the validated *Pfkelch13* mutants in the study areas suggests the absence of ART -R, although a significant proportion of D3 + cases were found. The absence of *dhfr/dhps* quintuple or sextuple mutants (quintuple + 581G) supports the continued use of SP for IPTp during pregnancy and in combination with amodiaquine for seasonal malaria chemoprevention.

*Trial registration*: ACTRN12623000344695.

**Supplementary Information:**

The online version contains supplementary material available at 10.1186/s12936-024-04922-1.

## Background

Malaria, a preventable and treatable disease, is estimated to cause 247 million cases and 619,000 deaths in 2021 [1]. The African region of the World Health Organization (WHO) accounts for 94% of global cases and deaths, with the burden concentrated in children under five years of age. Prompt and effective treatment of uncomplicated *Plasmodium falciparum* infections is the cornerstone of malaria control and elimination. Currently, the WHO recommends six artemisinin-based combinations for the treatment of uncomplicated falciparum malaria [2]: artemether-lumefantrine (AL), artesunate-amodiaquine (ASAQ), dihydroartemisinin-piperaquine (DP), artesunate-mefloquine (ASMQ), artesunate-sulfadoxine/pyrimethamine (ASSP), and artesunate-pyronaridine (AP).

*Plasmodium falciparum* resistance to anti-malarial drugs remains a common threat to effective case management and undermines global efforts to control and eliminate the malaria burden. Artemisinin partial resistance (ART -R), first described in Southeast Asia in 2008 [3, 4], is defined as delayed clearance (persistent parasitaemia at day 3-D3 +—or parasite clearance half-life > 5 h) after administration of artemisinin-based combination therapy (ACT) or artesunate monotherapy [5]. In vitro, ART-R is manifested by increased survival of early ring stage parasites (0–3 h post-invasion) after exposure to 700 nM dihydroartemisinin for 6 h [6, 7]. The emergence and spread of ART-R parasites in Southeast Asia was later followed by high rates of DP and ASMQ treatment failure associated with piperaquine and mefloquine resistance [8]. Molecular studies have confirmed the presence of non-synonymous mutations in *Pfkelch13* (PF3D7_1343700) as the primary determinant of ART-R [7, 9]. Currently, there are 13 validated (F446I, N458Y, M476I, Y493H, R539T, I543T, P553L, R561H, P574L, C580Y, C469Y, R622I, A675V) and nine candidate/associated (P441L, G449A, C469F, A481V, R515K, P527H, N537I/D, G538V, V568G) *Pfkelch13* mutations [6].

In sub-Saharan Africa, where most of the malaria burden occurs, the recent emergence and spread of validated *Pfkelch13* mutations in Rwanda (R561H), Uganda (A675V or C469Y), and the Horn of Africa (R622I) is of great concern [10–14]. This calls for frequent monitoring of the efficacy of first- and second-line ACT, including parasite clearance, and the prevalence of *Pfkelch13* mutations, as recommended by the WHO [5]. Increased copy numbers in the *plasmepsin II* (*pm2*) and *Plasmodium multi-drug resistance 1* (*Pfmdr-1*) genes have been associated with piperaquine [15] and mefloquine [16] resistance, respectively, resulting in decreased efficacy of DP [8, 17–21] and ASMQ [18, 22]. Although not yet validated [23], polymorphisms in the *chloroquine resistance transporter* (*Pfcrt*) and *Pfmdr-1* have been implicated in resistance to piperaquine, mefloquine, lumefantrine, and amodiaquine [24, 25]. In sub-Saharan Africa, the WHO also recommends chemoprevention to protect pregnant women with intermittent preventive treatment (IPTp) with sulfadoxine/pyrimethamine (SP) [26] and children using seasonal malaria chemoprevention (SMC) with sulfadoxine/pyrimethamine-amodiaquine (SPAQ) [27]. Polymorphisms in several codons in the genes encoding *dihydrofolate reductase* (*dhfr*) and *dihydropteroate synthase* (*dhps*) of *P. falciparum* are associated with an increased risk of SP treatment failure [28]. A quintuple mutation (51I, 59R, and 108N in the *dhfr* genes and 437G + 540E in the *dhps* genes) is a predictor of SP treatment failure [29, 30]. A sextuple mutant (quintuple + A581G) defined as super-resistant is associated with reduced efficacy of IPT-SP in pregnant women [31, 32] and infants [33].

Malaria is a major health problem in Togo, and the entire population is at risk, with children under five years of age being the most affected. Malaria transmission is stable, but with seasonal peaks from June to October in the north and from April to July and then from August to October in the south [34]. The estimated number of malaria cases and deaths in 2021 were 2.1 million and 3,715, respectively [1]. Since 2004, the Togolese National Malaria Control Programme (NMCP) has recommended AL and ASAQ for the treatment of uncomplicated *P. falciparum* infection [35]. Following the recommendation of SPAQ for seasonal malaria chemoprevention (SMC) in 2016, ASAQ was replaced by DP as second-line therapy for the treatment of uncomplicated falciparum infection [36]. The NMCP monitored the therapeutic efficacy of AL and ASAQ until 2013 and showed high efficacy with PCR-corrected cure rates of 96% or more for AL and 94% or more for ASAQ [35, 37].

The objectives of the present study were to evaluate the proportion of patients testing positive at day 3 (D3 +) and to assess the efficacy of AL and DP for the treatment of children with uncomplicated *P. falciparum* malaria. In addition, mutations in *Pfkelch13, Pfcrt, Pfmdr-1, dhfr, dhps* associated with *P. falciparum* resistance to anti-malarial drugs and deletions in *hrp2/hrp3* involved in variable performance of HRP2-based malaria rapid diagnostic tests (RDTs) were also investigated in isolates collected prior anti-malarial treatment.

## Methods

### Study design, population and areas

**Fig. 1:**
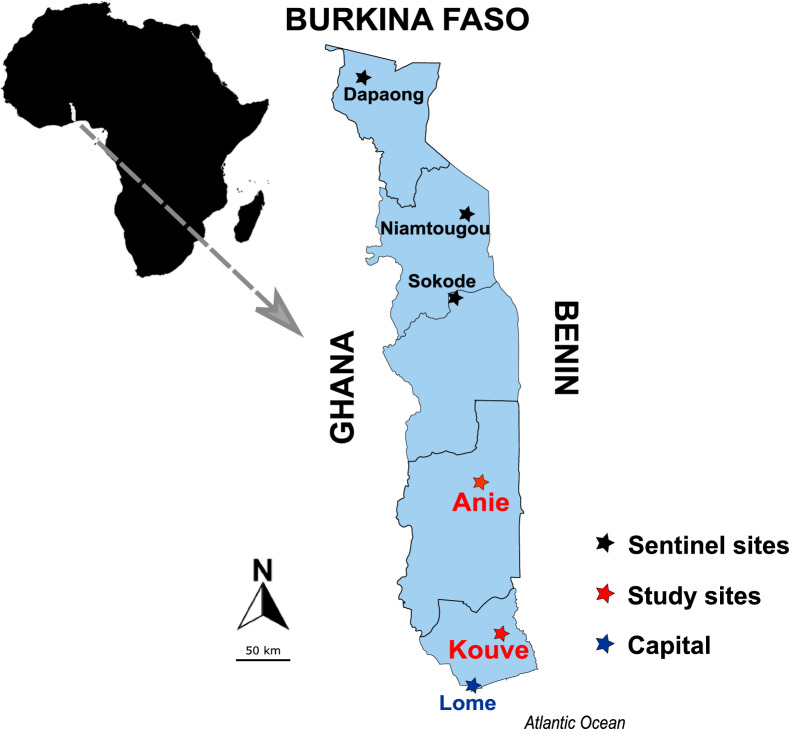
Map of Togo showing the current study sites

**T**his single arm prospective study evaluated the efficacy of AL or DP for the treatment of uncomplicated *P. falciparum* malaria in children aged 6–59 months. Eligible children were randomly assigned (not blinded) to receive either the AL or the DP. The study was conducted at the “La Providence” hospital in Kouvé for the Yoto District and the district hospital in Anié. The Kouvé site, located in the Maritime region, is approximately 76 km from the capital city (Lomé), and the Anié site, located in the Plateaux region, is 188 km from Lomé (Fig. 1).

## Recruitment

Patients attending the “La Providence hospital” in Kouvé and the Anié district hospital between 14 September 2021, and 29 January 2022, were screened for inclusion and exclusion criteria according to the WHO protocol [38]. After obtaining parental/guardian consent, children were enrolled if they were between 6 and 59 months of age, had an axillary temperature of ≥ 37.5 °C or a history of fever in the previous 24 h, had *P. falciparum* mono-infection with parasitaemia of at least 2,000 to 200,000 asexual parasites/µL based on microscopy, were able to take medicine orally, and agreed to be available for scheduled assessments during the study period. Children with severe falciparum malaria or danger signs according to the WHO definition [39], non-falciparum species or severe malnutrition, third-party administration of anti-malarial drugs, and non-malarial febrile illness were not enrolled, but received appropriate treatment according to national guidelines. Other exclusion criteria included body weight < 5 kg, haemoglobin level < 5 g/dl, regular medication interfering with anti-malarials pharmacokinetics, hypersensitivity to the test drugs, and treatment with AL or DP in the previous 48 h.

### Treatment and follow-up assessment

Recruited children were treated with either the AL or DP using random allocation sheets designed for this purpose. AL (20 mg artemether + 120 mg lumefantrine) was administered twice daily for three days based on the recommended weight bands: 1 tablet for 5–14 kg, 2 tablets for 15–24 kg, and 3 tablets for 25–34 kg). DP with a target daily dose of 4 mg/kg dihydroartemisinin + 18 mg/kg piperaquine for three days was administered as follows: ½ tablet (20/160 mg) for 5–6 kg, 1 tablet (20/160 mg) for 7–12 kg, 1 tablet (40/320 mg) for 13–23 kg, and 2 tablets (40/320 mg) for 24–35 kg. The study drugs were provided by the WHO and stored in a cool place. AL and DP were manufactured by Cipla Pharmaceutical Company LTD (India) and Fosun Pharma (China), respectively.

All treatment doses were administered under the direct supervision of the study nurse and observed for 30 min after administration. Any patient who vomited during the observation period was re-treated with the same dose of the test drug and observed for an additional 30 min. Children who vomited a second time were excluded from the study and were referred or admitted to the hospital, where the nationally recommended injectable artesunate was administered according to the national treatment guidelines. The parent/guardian of each recruited child enrolled was asked not to give any other medication during the follow-up period and to bring the child back if the child’s condition worsened.

Enrolled children were followed up for 42 days after the start of the treatment. Clinical and parasitological responses were monitored at scheduled visits on days 1, 2, 3, 7,14, 21, 28, 35, and 42, and on unscheduled days if the child’s condition worsened or symptoms recurred during the unscheduled days. The time window allowed for weekly follow-up was one day. Adverse events and severe adverse events, defined according to the WHO protocol for monitoring the therapeutic efficacy of anti-malarials [38], were clinically monitored at each follow-up visit. Adverse events were managed according to local practice as dictated by the pharmacovigilance department and the NMCP.

### Microscopy examination

Thick and thin blood smears were obtained from the fingerprick at day 0 and at follow-up visits. The blood slides were stained with Giemsa, and parasites were counted as the number of asexual parasites per 200 white blood cells (WBCs) or per 500 if the count was < 100 parasites/200 WBCs using the WHO method [38]. Each blood slide was examined independently by two qualified microscopists using Olympus CX23 microscope (Olympus Corporation, Tokyo, Japon), and the final parasitaemia was calculated by averaging the results of the two microscopists if they agreed (difference in parasite density < 50%). If the two counts differed by > 50% for parasite positivity, species, or density, the slides were re-examined by a third independent microscopist. For parasite species and positivity, two concordant results were considered the final result, whereas for parasite density, the average of the two closest estimates of parasitaemia was considered the final result. A blood slide was considered negative if no parasites were seen after counting 1000 WBCs. The presence of gametocytes at enrolment or follow-up was recorded. In addition, 100 fields of thick smears were examined on day 0 to exclude mixed infections. In cases of doubt, the thin film was examined for confirmation.

### Parasite genotyping

Filter paper blood samples (prepared from 2 to 3 drops of blood applied to Whatman #3 filter paper) were collected from each patient on day 0 and on the day of parasite recurrence (from day 7 onwards) and stored in individual plastic bags with desiccant until analysis, protecting them safe from light, moisture, and extreme temperatures. Each dried blood spot (DBS) was cut and placed in an Eppendorf tube. The 96-well protocol developed by Zainabadi et al*.* [40] was used to extract parasite DNA.

The eluted DNA was then quantified using fluorometric quantification (Thermo Fisher's Qubit) to 20 ng/µL and stored at − 20 °C for later use. According to recent WHO recommendation [41], paired DNA from patients with recurrent parasites (day 0 and day of recurrence) was genotyped using nested polymerase chain reaction (PCR) targeting the highly polymorphic genes *msp1* and *msp2*, and the microsatellite marker *poly-α*. All markers were systematically analysed. Capillary electrophoresis (Bioanalyzer, Agilent) was used to estimate fragment sizes, with cut-offs for PCR artifacts and stutter peaks set at 10% of the lower and upper control bands. The bins used to define a match were 10 bp for *msp1/msp2*, and 5 bp for *poly-α*. The genotyping data were compared with the previous genotyping approach for *msp1, msp2*, and *glurp* [42]. For *glurp*, the bin used to define a match was 20 bp. The WHO decision algorithm was used to calculate PCR-adjusted clinical efficacy rates. Recrudescence was defined as a genotype that had previously been detected in a blood sample taken prior to treatment (*i.e.,* at least one allele was shared at all three loci on day 0 and the day of parasite recurrence). A new infection was defined as the absence of a shared allele at any of the three loci between day 0 and the day of parasite recurrence.

### Markers of anti-malarial drug resistance

Day 0 DNA was examined for the presence of point mutations in the *Pfkelch13* gene (codons 430-720) associated with ART-R [3], the *Pfcrt* gene (codons 72-76, 93, 145, 218, 343, 350, and 353), and *Pfmdr-1* genes (codons 86, 184, 1034, 1042, and 1246) linked to 4-aminoquinolines and amino alcohol resistance [25] and the *dhfr* (codons 51, 59, 108, and 164) and *dhps* (codons 431, 436, 437, 540, 581, and 613) genes associated with pyrimethamine and sulfadoxine resistance [29]. Additionally, *hrp2* and *hrp3* gene deletions that may result in false-negative HRP2-based rapid diagnostic tests (RDTs) were examined [43].

As previously described [44], multiplex nested PCR assays with indexed primers with unique sample-specific tags (barcodes of individual 8-base indices) specific to the sample and adapter sequences (14 or 15 bases), which enabled the final PCR product to bind to the sequencing flow cell, were used to generated amplicons of targeted sequences were produced. To increase the sample volume and reduce the amount of sample for subsequent protocol steps, 4 µL of PCR reactions for each sample was pooled (96 samples). The amplicons were then purified for each pool using AMPure XP beads (Beckman Coulter), according to the manufacturer's instructions, to remove dNTPs, salts, primers, and primer dimers. The quality of the purified PCR products was assessed by analysing the eluates containing purified amplicons on a Bioanalyzer (Agilent). Fluorometric quantification (Qubit, Thermo Fisher) was used to determine the DNA concentration of the combined fragments. Prior to sequencing, the pooled libraries were denatured with 0.2 N NaOH, neutralized with 0.2 M Tris–HCL pH 7.5 before dilution with hybridisation buffer (HT1). Sequencing was performed using the MiSeq v2 reagent and the Illumina MiSeq Reagent kit v2 (300-cycles) according to the manufacturer’s instructions. Raw sequences were demultiplexed and quality reduced at a Phred score of 30. To avoid primer bias in the sequenced fragments, primer sequences were clipped from the 5′ end. Base calling was performed by comparing the reads to a custom database consisting of the 3D7 reference sequence. Bioinformatics analysis was carried out with the use of CLC Genomics Workbench 22 software (Qiagen). Laboratory reference parasite strains with known alleles for each gene (Dd2, 7G8, HB3, and a Cambodian strain 3601) were used as controls [13].

### Haemoglobin blood concentration

Blood haemoglobin concentration was measured on day 0 (prior to treatment) and on days 7, 14, 21, 28, 35, and 42 using a HemoCue Hb 301 apparatus (HemoCue AB, Ängelholm, Sweden) to assess the recovery of hAemoglobin levels.

### Classification of treatment outcome

Using WHO criteria [39], treatment outcomes were classified as an early treatment failure (ETF), late clinical failure (LCF), late parasitological failure (LPF) or adequate clinical and parasitological response (ACPR) before and after Polymerase Chain Reaction (PCR) correction. The primary endpoints were PCR-corrected per-protocol ACPR and Kaplan–Meier cure rates at day 28 and day 42 for AL and DP. The proportion of patients lost to follow-up and withdrawals was also calculated. Withdrawals included protocol violations, withdrawal of consent, failure to complete the study treatment (persistent vomiting, self- or third-party administration of anti-malarial drugs or antibiotics with anti-malarial activity, occurrence of concomitant disease during follow-up that would interfere with a clear classification of the treatment outcome, detection of mono-infection with another malaria species during follow-up, or misclassification of a patient due to a laboratory error (parasitaemia) leading to administration of rescue treatment).

### Sample size

A treatment failure rate of 5% for both AL and DP was assumed. A minimum sample size of 73 patients per site per drug was estimated at 95% confidence level with 5% precision. With a 20% increase to account for loss to follow-up and withdrawals during the 28-day or 42-day follow-up period, 88 patients per site per drug were targeted.

### Statistical analysis

Clinical and laboratory data for each patient were recorded on standard case record forms, double-entered independently, and analysed using a WHO Excel® database specifically designed for studies of anti-malarial drug efficacy (https://www.who.int/malaria/areas/drug_resistance/efficacy-monitoring-tools/en/).

Both per-protocol and Kaplan–Meier analyses were performed. For the per-protocol analysis, patients were excluded if they were lost to follow-up or withdrew during follow-up. In addition, patients with reinfection or indeterminate PCR results were excluded from the PCR-corrected per-protocol analysis. For the Kaplan–Meier analysis, patients who were lost to follow-up or withdrew during follow up were censored on the last day of follow-up, those with new infection were censored on the day of reinfection, and those with undetermined PCR were excluded. Baseline characteristics (age, sex, temperature, and parasitaemia) were compared between the two study sites according to the artemisinin-based combinations used for the treatment. Chi-square tests were used to compare categorical data. Differences in mean baseline age and parasite density were assessed using a two-sample Wilcoxon rank-sum (Mann–Whitney) test for non-normally distributed data. Gametocyte positivity rates at enrolment and follow-up were calculated. In addition, gametocyte clearance after treatment was assessed using Kaplan–Meier survival analysis. Confidence intervals were calculated for binomial proportions. Two-sided *p*-values of less than 0.05 were considered statistically significant.

### Ethical considerations

The study protocol was approved by the Committee of Bioethics for Research in the Health of Togo. Parents or caregivers of the children were informed of the aims and procedures of the study and provided written informed consent. If a parent or caregiver was illiterate, they chose a witness to sign the consent form. All children enrolled in the study received free care throughout the follow-up period. All hospitalization costs were covered by the project. Travel expenses for scheduled and unscheduled visits were reimbursed and each child received treated mosquito bed nets. Community leaders in the areas surrounding the study sites were informed of the study objectives and procedures.

## Results

### Socio-demographic profile of the study children

Of the 741 children screened (343 in Anié and 398 in Kouvé) between September 2021 and January 2022, 165 (82 for AL and 83 for DP) in Anié and 192 (97 for AL and 95 for DP) in Kouvé were eligible and enrolled in the study (Table 1). The baseline characteristics of the different groups were comparable, except for parasitaemia. Patients in Kouvé had significantly higher parasitaemia compared to Anié for both AL and DP treatment groups (p < 0.0001 between AL groups; p < 0.0001 for AL vs. DP groups; and p < 0.0001 between the DP treated groups). Quality control of the blood slides by an experienced microscopist revealed that 20 children had parasitaemia above the upper limit of 200,000 asexual parasites/microlitre of blood on day 0 according to the protocol. Of these, three cases were in the DP group in Anié, 11 in the DP group in Kouvé, and 6 in the AL group in Kouvé. There was no significant difference between mean Hb levels on day 0 of children with parasitaemia ≥ 200,000 asexual parasites/microlitre and those above this threshold within each treatment group (p = 0.2 for Anié DP, p = 0.5 for Kouvé Al, and p = 0.7 for Kouvé DP).

**Table 1:** Baseline characteristics of the study patients by site and treatment groups, Togo, 2021–2022

Characteristics	Kouvé		Anié	
	AL^a^	DP^b^	AL^a^	DP^b^
Number of patients	97	95	82	83
Males (%)	44/97 (45.4)	49/95 (51.6)	38/82 (46.4)	49/83 (59.0)
Age (years)				
Mean (± SD^c^)	2.9 (± 1.3)	2.9 (± 1.3)	3 (± 1.3)	2.8 (± 1.3)
Range (min–max)	0.5–4.9	0.6–4.9	0.5–4.9	0.5–4.9
Temperature (°C)				
Mean (± SD^c^)	38.4 (± 1.2)	38.5 (± 1.3)	38.1 (± 0.7)	38 (± 0.7)
Haemoglobin level (g/dL)				
Mean (± SD^c^)	10.3 (± 1.6)	9.8 (± 1.8)	10.0 (± 1.4)	9.4 (± 1.7)
Parasitaemia (/µL), day 0				
Geometric mean	38,161*	39,016**	14,527*	9,828**
Range (min–max)	2,088–363,230	2,004–548,400	2,114–184,667	2,024–339,000

^a^artemether-lumefantrine,

^b^dihydroartemisinin-piperaquine,

^c^standard deviation

*Patients in Kouvé had significantly higher parasite density compared to Anié for AL (p < 0.0001) treated groups

**Patients in Kouvé had significantly higher parasite density compared to Anié for DP (p < 0.0001) treated groups

### Treatment outcome

Table 2 summarizes the unadjusted PCR outcomes at days 28 and 42 based on per-protocol and Kaplan–Meier analysis. At day 28, the per-protocol PCR-uncorrected cure rates for AL were 88.8% and 88.6% for Kouvé and Anié, respectively, and for DP were 98.9% and 97.4% for Kouvé and Anié, respectively. At day 42, the PCR-uncorrected cure rates for AL were 82.0% and 83.5% for Kouvé and Anié, respectively, while the cure rates for DP were 92.2%, and 94.7%, for Kouvé and Anié, respectively. Similar rates were observed in the Kaplan–Meier analysis (Table 2). Most recurrences were observed in the AL-treated group. In Kouvé, 10 patients (AL) and 1 patient (DP) experienced parasite recurrence on day 28. Similarly, in Anié, 9 patients (AL) and 2 patients (DP) had parasite recurrence on day 28. y day 42, the number of patients with parasite recurrence was 16 patients (AL) and 7 patients 4 patients (DP) had parasite recurrence in Anié.

**Table 2:** PCR uncorrected treatment responses for artemether-lumefantrine or dihydroartemisinin-piperaquine by day 28 and 42 days

PCR-uncorrected Outcome	Lost/ withdrawn	Per-protocol analysis								Kaplan Meier Cure rate
		ETF^a^		LCF^b^		LTF^c^		ACPR^d^		
	No (%)	No	% (CI 95%)	No	% (CI 95%)	No	% (CI 95%)	No	% (CI 95%)	% (CI 95%)
Kouvé site (Day 28)										
AL^e^ (n = 97)	8 (8.2)	0	0.0 (0.0–4.1)	4	4.5 (1.2–11.1)	6	6.7 (2.5–14.1)	79	88.8 (80.3–94.5)	88.9 (80.4–93.9)
DP^f^ (n = 95)	5 (5.3)	0	0.0 (0.0–4.0)	0	0.0 (0.0–4.0)	1	1.1 (0.0–6.0)	89	98.9 (94.0–100)	98.9 (92.7–99.8)
Anié site (Day 28)										
AL^e^ (n = 82)	3 (3.7)	0	0.0 (0.0–4.6	4	5.1 (1.4–12.5	5	6.3 (2.1–14.2)	70	88.6 (79.5–94.7)	88.6 (79.3–93.9)
DP^f^ (n = 83)	7 (8.4)	0	0.0 (0.0–4.7)	1	1.3 (0–7.1)	1	1.3 (0–7.1)	74	97.4 (90.8–99.7	97.4 (89.9–99.3)
Kouvé site (Day 42)										
AL^e^ (n = 97)	8 (8.2)	0	0.0 (0.0–4.1)	6	6.7 (2.5–14.1)	10	11.2 (5.5–19.7)	73	82.0 (72.5–89.4)	82.2 (72.6–88.7)
DP^f^ (n = 95)	6 (6.3)	0	0.0 (0.0–4.1)	2	2.2 (0.3–7.9)	5	5.6 (1.8–12.6)	82	92.1 (84.5–96.8)	92.3 (84.5–96.2)
Anié site (Day 42)										
AL^e^ (n = 82)	3 (3.7)	0	0.0 (0.0–4.6)	5	6.3 (2.1–14.2)	8	10.1 (4.5–19.0)	66	83.5 (73.5–90.9)	83.5 (73.4–90.1)
DP^f^ (n = 83)	7 (8.4)	0	0.0 (0.0–4.7)	1	1.3 (0.0–7.1)	3	3.9 (0.8–11.1)	72	94.7 (87.1–98.5)	94.7 (86.6–98.0)

^a^early treatment failure

^b^late clincal failure

^c^late parasitological failure

^d^adequate clinical and parasitological response

^e^artemether-lumefantrine

^f^dihydroartemisinin-piperaquine

Of the 40 recurrences detected at day 42, 6 were found to be recrudescent infections (true treatment failure), 5 gave non-conclusive genotyping results (3 in Kouvé and 2 in Anié of the AL-treated groups), and 29 were classified as new infections (based on the genotyping approach using nested polymerase chain reaction (PCR) targeting the highly polymorphic genes *msp1* and *msp2*, and the microsatellite marker *poly-α* and the WHO/MMV match-counting algorithm (3/3), as recommended by the WHO) [42]. Details are shown in Additional file 1: Table S1.

On day 28, per-protocol PCR-corrected cure rates of 97.5% and 98.6% were observed for AL in Kouvé and Anié, respectively, while children treated with DP achieved a 100% cure rates in both sites (Table 3). At day 42, PCR-corrected AL cure rates of 96.1% and 97.1% were observed in Kouvé and Anié, respectively, and PCR-corrected DP cure rates of 98.8% and 100% were observed in Kouvé and Anié, respectively. Kaplan–Meier analysis showed almost similar cure rates (Table 3). Parasite persistence on day 3 (D3 +) in Anié were detected in 7/82 patients (8.5%, CI 95%: 3.5–16.8%) treated with AL and 2/76 patients (2.6%, CI 95%: 0.3–9.2%) treated with DP. The difference in proportions between AL and DP groups was not significant (p = 0.17, Fisher’s exact test). In Kouvé, 4/93 patients (4.3%, CI 95%: 1.2–10.6%) treated with AL and 2/94 patients (2.1%, CI 95%: 0.3–7.5%) treated with DP had parasites on day 3. Again, the difference in proportions was not significant (p = 0.44, Fisher’s exact test). Parasite densities found on day 3 were very low across all sites, ranging from 11 to 72 asexual parasites per microlitre. Out of the 20 cases with day 0 parasitaemia ≥ 200,000 asexual parasite/microlitre of blood, 19 were parasite free at day 3 and achieved ACPR. The remaining case was parasite positive on day 3 and had recrudescence at day 42.

**Table 3:** PCR-corrected treatment responses for artemether-lumefantrine or dihydroartemisinin-piperaquine by day 28 and 42 days

PCR-corrected Outcome	Lost/ withdrawn	Reinfection/Unknown^a^	Per-protocol analysis							Kaplan Meier Cure rate
			Total analysable	LCF^b^		LTF^c^		ACPR^d^		
	No(%)	No(%)		No	%(CI 95%)	No	%(CI 95%)	No	%(CI 95%)	%(CI 95%)
Kouvé site (Day 28)										
AL^e^ (n = 97)	8(8.2)	8(8.2)	81	1	1.2(0.0–6.7)	1	1.2(0.0–6.7)	79	97.5(91.4–99.7)	97.8(91.3–99.4)
DP^f^ (n = 95)	5(5.3)	1(1.1)	89	0	0.0(0.0–4.1)	0	0.0(0.0–4.1)	89	100(95.9–100)	100
Anié site (Day 28)										
AL^e^ (n = 82)	3(3.7)	8(9.8)	71	1	1.4(0.0–7.6)	0	0.0(0.0–5.1)	70	98.6(92.4–100)	98.7(91.2–99.8)
DP^f^ (n = 83)	7(8.4)	2(2.4)	74	0	0.0(0.0–4.9)	0	0.0(0.0–4.9)	74	100(95.1–100)	100
Kouvé site (Day 42)										
AL^e^ (n = 97)	8(8.2)	13(13.4)	76	1	1.3(0.0–7.1)	2	2.6(0.3–9.2)	73	96.1(88.9–99.2)	96.4(89.1–98.8)
DP^f^ (n = 95)	6(6.3)	6(6.3)	83	0	0.0(0.0–4.3)	1	1.2(0.0–6.5)	82	98.8(93.5–100)	98.9(92.1–99.8)
Anié site (Day 42)										
AL^e^ (n = 82)	3(3.7)	11(13.4)	68	1	1.5(0.0–7.9)	1	1.5(0.0–7.9)	66	97.1(89.8–99.6)	97.3(89.5–99.3)
DP^f^ (n = 83)	7(8.4)	4(4.8)	72	0	0.0(0.0–5.0)	0	0.0(0.0–5.0)	72	100(95.0–100)	100

^a^samples with inconclusive PCR result: 3 in Kouvé and 2 in Anié AL treatment groups

^b^late clincal failure

^c^late parasitological failure

^d^adequate clinical and parasitological response

^e^artemether-lumefantrine

^f^dihydroartemisinin-piperaquine

Of the 357 children included in the cohorts, 35 (9.8%) reported mild adverse reactions on days 1 and 2: 16 with vomiting, 18 with weakness and 1 case with anorexia. Vomiting was more frequent in the DP group (6.2%, 11/179) than in the AL group (2.8%, 5/178), but the difference was not significant (p = 0.13, Fisher exact test).

### Haemoglobin recovery

There was a significant progressive increase in mean haemoglobin levels during the follow-up visits except at day 7 for Kouvé where no significant increase or even a significant decrease was observed (Table 4). There was no significant difference between mean Hb levels of children with parasitaemia ≤ 200,000 and those above this threshold except for Kouvé DP group at day 14 (Hb day14 of 10.5 g/dl vs 9.3 g/dl, p = 0.01, respectively).

**Table 4:** Haemoglobin increase (compared to Hb D0) during the follow period among children treated with artemether-lumefantrine (AL) or dihydroartemisinin-piperaquine (DP) at Kouvé and Anié. Togo. 2021–2022

Drug/Site	Analysis	Hb D7	Hb D14	Hb D21	Hb D28	Hb D35	Hb D42
AL^a^							
Anié	Mean increase	0.29	0.88	1.02	1.22	1.23	1.31
	95% CI	0.03–0.55	0.58–1.19	0.72–1.32	0.90–1.53	0.90–1.57	0.95 to 1.67
	*P-value*	< *0.04*	< *0.0001*	< *0.0001*	< *0.0001*	< *0.0001*	< *0.0001*
Kouvé	Mean increase	0.05914	0.91	1.4811	1.71	1.74	1.84
	95% CI	− 0.21 to 0.33	0.60 to 1.23	1.12 to 1.84	1.33 to 2.09	1.36 to 2.14	1.44 to 2.24
	*P-value*	*0.66*	< *0.0001*	< *0.0001*	< *0.0001*	< *0.0001*	< *0.0001*
DP^b^							
Anié	Mean increase	0.46	1.14	1.57	1.65	1.76	1.82
	95% CI	0.10 to 0.81	0.80 to 1.48	1.22 to 1.93	1.27 to 2.04	1.36 to 2.15	1.46 to 2.18
	*P-value*	< *0.02*	< *0.0001*	< *0.0001*	< *0.0001*	< *0.0001*	< *0.0001*
Kouvé	Mean decrease	− 0.3284	0.58	1.18	1.51	1.62	1.74
	95% CI	− 0.59 to − 0.07	0.23 to 0.93	0.23 to 0.93	1.16 to 1.86	1.25 to 1.98	1.25 to 1.98
	*P-value*	< *0.02*	< *0.002*	< *0.0001*	< *0.0001*	< *0.0001*	< *0.0001*

^a^artemether-lumefantrine

^b^dihydroartemisinin-piperaquine

### Molecular markers of anti-malarial drug resistance

All 357 day 0 samples were successfully analysed for mutations in the *Pfkelch13, Pfcrt, Pfmdr-1*, *dhfr*, and *dhps* genes*.* Details are shown in Table 5 and 6. Of these, 99.2% (354/357) carried the *Pfkelch13* wild-type allele. Two isolates from Kouvé carried the A578S mutation, which is known not to confer resistance in vitro, and one isolate from Kouvé carried the A557S mutation, which is a non-validated *Pfkelch13* mutation.

**Table 5:** Number and frequency of SNPs detected in genes associated with anti-malarial drug resistance in day0 samples by study site, Togo, 2021–2022

Gene	Codon	AA	No. of sample			% of mutant			p-value
			Anié	Kouvé	Total	Anié	Kouvé	Total	
*Pfkelch13*	WT	–	165	189	354	0.0%	1.6%	1.6%	0.3
	557	A > S	0	1	1				
	A578	A > S	0	2	2				
*Pfcrt*	72	C	165	191	356	0.0%	0.5%	0.3%	1
		S	0	1	1				
	74	M	164	186	350	0.6%	3.1%	2.0%	0.12
		I	1	6	7				
	75	N	164	186	350	0.6%	3.1%	2.0%	0.12
		E	1	6	7				
	76	K	164	185	349	0.6%	3.6%	2.2%	0.07
		T	1	7	8				
	356	I	165	185	350	0.0%	3.6%	2.0%	**0.01**
		T	0	7	7				
*Pfmdr-1*	86	N	164	192	356	0.6%	0.0%	0.3%	0.46
		Y	1	0	1				
	184	Y	43	43	86	73.9%	77.6%	75.9%	0.45
		F	122	149	271				
*dhfr*	51	N	7	12	19	95.8%	93.8%	94.7%	0.48
		I	158	180	338				
	59	C	0	1	1	100.0%	99.5%	99.7%	1
		R	165	191	356				
	108	S	64	20	84	61.2%	89.6%	76.5%	** < 10**^**–6**^
		N	101	172	273				
*dhps*	431	I	156	182	338	5.5%	5.2%	5.3%	1
		V	9	10	19				
	436	S	74	104	178	55.2%	45.8%	50.1%	0.08
		A	91	88	179				
	437	G	157	188	345	4.8%	2.1%	3.4%	0.24
		A	8	4	12				
	540	K	165	190	355	0.0%	1.0%	0.6%	0.5
		E	0	2	2				
	581	A	163	184	347	1.2%	4.2%	2.8%	0.11
		G	2	8	10				
	613	A	148	169	317	10.3%	12.0%	11.2%	0.73
		S	17	23	40				

P values are in bold fonts when the value is less than 0.05, meaning that the proportions tested are significantly different

**Table 6:**
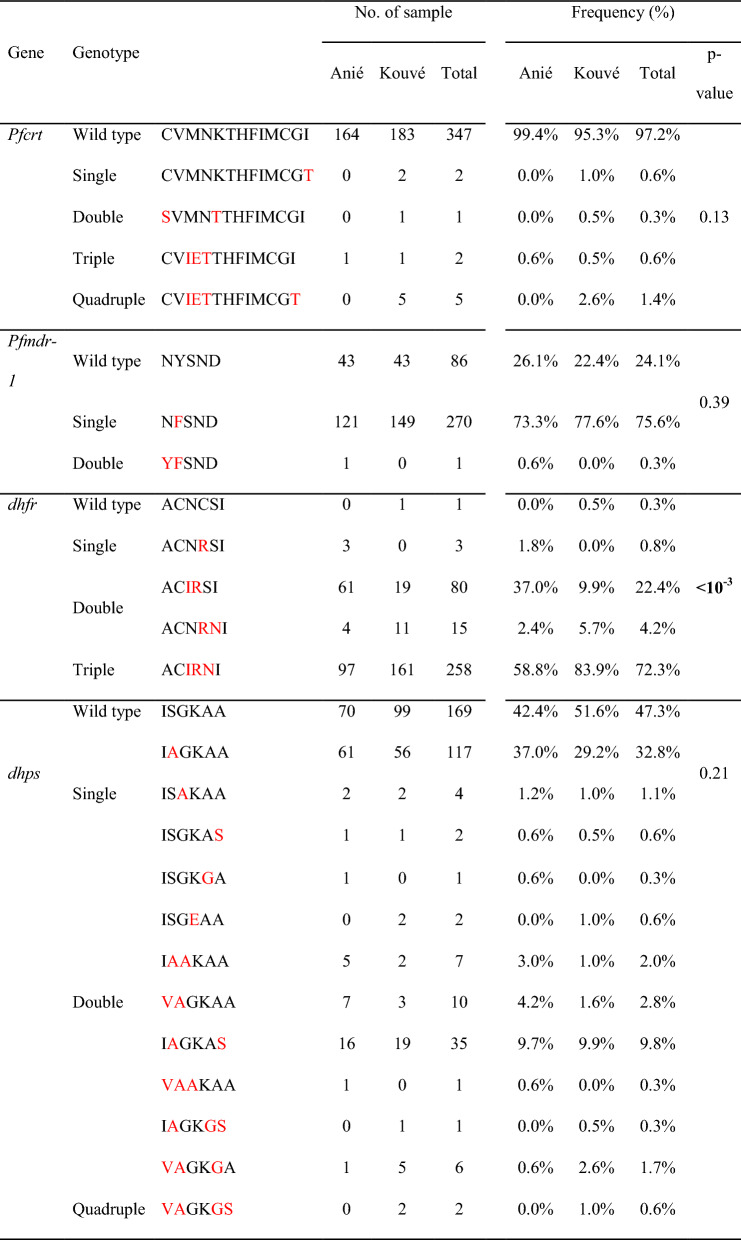
Number and frequency of different genotypes detected in genes associated with anti-malarial drug resistance from day0 samples by study site, Togo, 2021–2022

P values are in bold fonts when the value is less than 0.05, meaning that the proportions tested are significantly different.

Position of amino acid corresponds to codons 72, 73, 74, 75, 76, 93, 97, 145, 218, 343, 350, 353 and 356 for pfcrt, to codons 86, 184, 1034, 1042 and 1246 for pfmdr-1, to codons 16, 50, 51, 59, 108, 164 for dhfr and to codons 431, 436, 437, 540, 581, 613 for dhps. Amino acid changes are shown in red

For *Pfcrt,* five mutations were identified, and five different alleles were detected, of which 97.2% were wild type. The most frequent other parasites were those carrying the 74I/75E/76 T/356 T allele (1.4%), followed by those carrying the 74I/75E/76 T allele (0.6%), the 356 T allele (0.6%), and the 72S/76 T allele (7G8 allele, 0.3%). It is noteworthy that a significant difference in proportion was observed between sites for the I356T mutation, which was only detected in Kouvé (3.6% in Kouvé vs. 0% in Anié, p = 0.01).

For *Pfmdr-1*, two mutations were found (N86Y and 184F) and three alleles were identified, including the 184F single mutant (75.6%), the wild-type allele (24.1%), and the 86Y/184F double mutants (0.3%). Similar proportions of mutations were found between the two sites.

Three mutations (N51I, C59R and S108N) and five different *dhfr* alleles were detected in the tested isolates. These included the triple 51I/59R/108N mutant allele (72.3%), the double 51I/59R mutant allele (22.4%), the double 59R/108N mutant allele (4.2%), the single 59R mutant allele (0.8%), and the wild-type allele (0.3%). Significant differences in proportions were observed for the S108N mutation (61.2% in Kouvé vs. 89.6% in Anié, p < 10^–6^) and for the distribution of the *dhfr* alleles between sites (p < 0.0001).

For *dhps*, 6 mutations (I431V, S436A, G437A, K540E, A581G and A613S) and 12 alleles were found. The most common allele was the wild-type allele (47.3%). The single 436A mutant allele was found in 32.8% of the isolates tested, followed by the double 436A/613S mutant allele (9.8%), the double 431 V/436A mutant allele (2.8%), and the double 436A/437A mutant allele (2.0%). The 540E and 581G mutations were observed in two and ten isolates, respectively. No significant difference in frequency was observed.

Combining the two *dhfr/dhps* loci without estimating the complexity of infection allowed the frequency of the *dhfr/dhps* haplotypes to be inferred. Twenty-five haplotypes were found. The most frequent *dhfr/dhps* haplotype was the triple 51I/59R/108N mutant (33.3%), followed by the quadruple 51I/59R/108N + 436A mutant (24.6%), the double 51I/59R mutant (10.6%), the triple 51I/59R + 436A mutant (7.3%), and the quintuple 51I/59R/108N + 436A/613S mutant (7.3%). The 59R/108N double mutant (2.5%) and the 51I/59R + 436A/613S quadruple mutant (2.2%) were found in proportions of < 10%. The 51I/59R/108N + 437A/540E quintuple mutant, which is associated with SP treatment failure in adults and children, was not detected.

### *hrp2/hrp3* genetic deletions

Genetic deletions in the *hrp2* and *hrp3* genes in the Togolese *P. falciparum* population were also sought, as there are two important factors responsible for the variable performance of malaria RDTs. For this analysis, 357 day 0 samples were tested. Only one isolate with *hrp2* deletion and one with *hrp3* deletion were found in Kouvé (0.3%) and Anié (0.3%), respectively. Dual *hrp2/hrp3* deletions, which could potentially compromise the efficacy of HRP2-based RDTs, were not observed.

## Discussion

This study shows that AL, the recommended first-line treatment in Togo, is achieving high cure rates (PCR-corrected cure rates of 97.8% in Kouvé and 98.7% in Anié on day 28 and 96.4% in Kouvé and 97.3% in Anié on day 42, respectively), eight years after the last clinical efficacy study, which was completed in 2013 [37]. The study also assessed, for the first time, the efficacy of DP, a second-line treatment introduced in 2016, and demonstrated high efficacy (PCR corrected cure rates of 100% in Kouvé and Anié on day 28 and 98.9% in Kouvé and 100% in Anié on day 42, respectively). These results are similar to those reported in neighboring countries such as Ghana [45] and Benin [46], but also as well as other West African countries [47–54], and Central and East African countries [55–57]. This contrasts with recent reports of the reduced PCR-corrected cure rates of AL (< 90%) found, in the absence of a *Pfkelch13* validated variants, in Burkina Faso [58], Angola [59], and the Democratic Republic of Congo [60], suggesting lumefantrine resistance. In the Burkina Faso study, Rasmussen and Ringwald [61] noted some methodological deviations from the WHO standard procedure for assessing therapeutic efficacy on which the reported treatment outcome classifications are based. Similarly, studies in Angola and DRC used microsatellite-based PCR correction and Bayesian algorithm and not the WHO-recommended method for PCR correction [42]. The reported reduced efficacy of AL treatment is of great concern and calls for confirmatory studies in which lumefantrine blood levels are measured in addition to TES to ensure adequate blood levels of the drug [62]. In addition to parasite resistance, other factors affecting treatment outcomes, include suboptimal blood drug levels due to incorrect dosing, poor patient compliance, poor drug quality, and drug-drug interactions. Notable challenges include the difficulty of monitoring the second AL dose (evening) when patients are not hospitalized and the need for co-administration with fatty foods to improve lumefantrine absorption.

Currently, TES is the gold standard for monitoring anti-malarial drug efficacy and is the WHO standard on which the current treatment outcome grading and the 90% threshold for the treatment policy change are based [38]. TES is conducted in a controlled environment where drug administration is monitored, the results of microscopic examination of blood films are validated, and the origin and quality of drugs are verified. Therefore, adherence to the standard WHO-recommended protocol for monitoring the efficacy of first- and second-line treatments is critical to mitigate these patient-level cofactors of treatment response and to generate reliable data.

Molecular investigation of the Togolese isolates did not reveal any validated *Pfkelch13* variant, suggesting a lack of artemisinin partial resistance. Only 2 isolates in Kouvé carried the A578S mutation, which is not known to confer resistance in vitro, and one isolate in Kouvé carried the A557S mutation, which is a non-validated *Pfkelch13* mutation. Although the parasite densities found on day 3 were very low at all sites (ranging from 11 to 72 asexual parasites per microlitre), the parasite positivity rate at day 3 of up to 8.5% is of concern and require close monitoring as a positivity rate of more than 10% on day 3 after treatment initiation is a clinical surrogate marker for suspected artemisinin partial resistance [5]. This is particularly emphasized as recent reports of validated *Pfkelch13* mutations with delayed parasite clearance after ACT treatment have been observed in Rwanda [10], and Uganda [11] and Eritrea [14] underlining the importance of continued monitoring of anti-malarial drug efficacy and resistance to artemisinins and partner drugs.

Analysis of the isolates to search for mutations in the *Pfcrt* gene showed that most of the parasites carried the *Pfcrt* wild-type allele (95.3% in Anié and 99.4% in Kouvé). The *Pfcrt* variant associated with resistance to chloroquine (CVIET) was detected in two isolates (one in Anié and one in Kouvé). This proportion is relatively low compared to other regions such as Democratic Republic of the Congo [63], Equatorial Guinea [64], and Liberia [54]. Notably, the 7G8 allele, which is predominant in South America, was detected in one isolate from Kouvé. For the *Pfmdr-1* gene, the single 184F mutation, which has no clear impact on ACT drug efficacy, was highly prevalent in Togo (75.9%). This is usually reported in many African settings such as Ghana [65], Uganda [66], Madagascar [67], Tanzania [68], and Equatorial Guinea [69], Chad [44],

Molecular analysis looking for mutations in the *dhfr* gene revealed the virtual absence of the *dhfr* wild-type allele (0.3%, found in one isolate at Kouvé) contrasting with the near saturation of the N51I and the C59R mutations, with proportions estimated at 94.7% and 99.7%. However, the proportion of the S108N mutation was found at a lower frequency (76.5%) and varied significantly between sites (p < 10^–6^). For the *dhps* gene, the S436 mutation was dominant, reaching a mean of 50.1%. The other mutations were found in a proportion < 10%, except for the A613S mutation. Although, the complexity of infection was not estimated, the frequency of the *dhfr/dhps* haplotypes was inferred by combining the most predominant mutations found in the two *dhfr/dhps* loci*.* The most frequent *dhfr/dhps* haplotype was the 51I/59R/108N triple mutant (33.3%), followed by the 51I/59R/108N + 436A quadruple mutant (24.6%). Sulfadoxine/pyrimethamine is used for intermittent preventive treatment of malaria in pregnancy and in combination with amodiaquine in SMC to reduce the burden of malaria in the most vulnerable groups (pregnant women and children) in Togo, as recommended by the WHO [26, 27]. Mutations in the *dhfr* and *dhps* genes are markers of SP resistance, and quintuple *dhfr/dhps* mutations (N51I/C59R/S108N-A437G/K540E) have been associated with clinical treatment failure with SP [28, 29]. In addition, reduced efficacy of IPT-SP in infants and pregnant women has been observed in areas where parasites with the sextuple mutation (quintuple + 581G) are present [70, 71]. The absence of the quintuple mutation 51I/59R/108N + 437G/540E in the current study in Togo is encouraging and consistent with other West African countries where this mutation is absent or present at very low levels [54, 72–78], compared to the high frequency (> 70%) observed in East Africa [79–83]. Given the use of SP as part of chemoprevention strategies in Togo, the drug will continue to exert pressure on the parasites and therefore resistance markers of SP should be continuously monitored.

This study also provides, for the first time, an estimate of the proportion of *hrp2*, *hrp3* and dual *hrp2/3* gene deletions in Togo which could be used as baseline data for future surveys to determine the trend in the frequency of *hrp2/3* gene deletions. Of the 357 isolates tested, only one isolate with an *hrp2* deletion and one with an *hrp3* deletion were found only in Kouvé. Dual *hrp2/hrp3* deletions, which could compromise the efficacy of HRP2-based RDTs, were not observed [84, 85]. Similar to the proportions usually observed in African countries, this finding validates the continued used of HRP2-based RDTs in Togo.

Malaria is the leading cause of anemia in children under five living in areas of high transmission due to repeated malaria infections [86]. Nutritional deficiencies, helminth infections, and haemoglobinopathies also cause anemia in Africa [87, 88]. The results of the current study show a significant increase in Hb levels after treatment. In addition to prompt effective treatment to resolve the acute infections, prevention of repeated infections through chemoprevention and vector control measures (long-lasting insecticidal nets, insecticide residual spraying) is critical to control malaria-related anaemia [89].

## Conclusion

The results of this study confirm that first-line (AL) and second line (DP) treatments are highly effective in treating uncomplicated falciparum infections, with a cure rate of over 96%. The absence of validated *Pfkelch13* mutants in the study sites suggests the absence of ART-R. The absence of *dhfr/dhps* quintuple and quintuple + 581G sextuple mutations supports the continued use of SP for IPTp during pregnancy. However, routine monitoring of anti-malarial drug efficacy and resistance should continue in Togo to detect any changes in the susceptibility of the parasite populations.

### Supplementary Information

**Additional file 1: Table S1.** Raw data of *msp-1, msp-2*, *glurp* and *poly α* polymorphisms (band size in bp) detected on day0 and day of recurrence (dayX) in isolates from recurrent infections, Togo, 2021-2022.

## Data Availability

The dataset used in this study is available and can be shared upon reasonable request with NMCP through the corresponding author.

## Abbreviations

ACPR: Adequate clinical and parasitological response
ACTs: Artemisinin-based combination therapies
AL: Artemether-lumefantrine
SPAQ: Sulfadoxine/pyrimethamine-amodiaquine
ART-R: Artemisinin resistance
ASAQ: Artesunate-amodiaquine
ASMQ: Artesunate-mefloquine
ASPY: Artesunate-pyronaridine
ASSP: Artesunate-sulfadoxine/pyrimethamine
*dhps*: dihydropteroate synthetase
DP: Dihydroartemisinin-piperaquine
ETF: Early treatment failure
LCF: Late clinical failure
LPF: Late parasitological failure
*msp1*: Merozoite surface proteins 1
*msp2*: Merozoite surface proteins 2
*glurp*: Glutamate rich-protein
PCR: Polymerase chain reaction
*dhfr*: Dihydrofolate reductase-thymidylate synthase
Pfmdr-1: Plasmodium falciparum multi drug resistance 1
Pfkelch13: Plasmodium falciparum kelch13
SMC: Seasonal malaria chemoprevention
WHO: World Health Organization
