# Supplementary material for: Efficacy of artemether-lumefantrine and dihydroartemisinin-piperaquine and prevalence of molecular markers of anti-malarial drug resistance in children in Togo in 2021
Source: Malar J. 2024 Apr 3;23:92. doi: 10.1186/s12936-024-04922-1 (PMC10988893; doi:10.1186/s12936-024-04922-1)
Supplement: Supplementary file 1 — Additional file 1: Table S1. Raw data of msp-1, msp-2, glurp and poly α polymorphisms (band size in bp) detected on day0 and day of recurrence (dayX) in isolates from recurrent infections, Togo, 2021-2022. [file 12936_2024_4922_MOESM1_ESM.docx]

**Table S1.** Raw data of *msp-1, msp-2*, *glurp* and *poly α* polymorphisms (band size in bp) detected on day0 and day of recurrence (dayX) in isolates from recurrent infections, Togo, 2021-2022.

| **ID** | **Day0** | **DayX** | ***msp1*** | | | | | | ***msp2*** | | | | ***glurp*** | | ***poly α*** | |
| --- | --- | --- | --- | --- | --- | --- | --- | --- | --- | --- | --- | --- | --- | --- | --- | --- |
|  |  |  | **K1**  **D0** | **K1**  **DX** | **RO33 D0** | **RO33 DX** | **Mad20 D0** | **Mad20 DX** | **3D7**  **D0** | **3D7 DX** | **FC27 D0** | **FC27 DX** | **glurp D0** | **glurp DX** | **poly α**  **D0** | **poly α DX** |
| 06TG046 | Day0 | Day28 | 164/211 | - | 131 | - | 193/219 | 184 | - | - | 476 | - | 829 | 800 | 186 | 172 |
| 06TG047 | Day0 | Day28 | 175 | 220 | 136 | 136 | - | - | - | 289 | 395 | 488 | 810 | 744 | 186 | 159 |
| 06TG048 | Day0 | Day28 | 178 | - | 136 | 138 | - | 139 | 260 | 294 | 363 | 330 | - | - | 164 | 188 |
| 06TG058 | Day0 | Day28 | 228 | 207 | 138 | - | 253 | - | 238 | 262 | 375 | - | 642 | - | 202 | 195 |
| 06TG070 | Day0 | Day21 | 173/200 | 152/178 | 142 | - | 218 | 214 | 257 | 270 | 360 | 363 | 693 | 729 | 205 | 221 |
| 06TG114 | Day0 | Day35 | 149 | - | - | - | 153 | - | 212 | - | 392 | - | 792 | - | 181 | - |
| 06TG153 | Day0 | Day21 | 161 | 186 | 143 | 145 | 237 | 228 | 235/262 | 272 | 360 | 359/426 | 566/729 | 426/817 | 214 | 213 |
| 06TG159 | Day0 | Day35 | 186 | 144 | 145 | 148 | 200 | 196 | 278 | 170 | 363/435 | 355/435 | 784 | 722 | 215 | 189 |
| 06TG184 | Day0 | Day42 | 173 | 200 | 145 |  | 232 | - | 247 | - | 367/444 |  | 737 | 550 | 177 | 172 |
| 06TG186 | Day0 | Day35 | 163 | 145 | 148 | - | 221 | - | 218 | - | - | - | 835 | 784 | 184 | 176 |
| 06TG189 | Day0 | Day28 | 186 | - | 131 | - | 221 | - | 189 | 267 | 383 | - | 680 | 667 | 187 | 162 |
| 06TG195 | Day0 | Day21 | - | 218 | 150 | - | 158/222 | 159 | 236 | - | 375 | - | 618 | - | 192 | - |
| 06TG212 | Day0 | Day28 | 186 | 211 | 135 | - | 164 | 245 | 260 | 236 | 379/459 | 404 | 667 | 540 | 141 | 171 |
| 06TG287 | Day0 | Day21 | 181 | - | 150 | 153 | 163/217 | 163/262 | 252 | 262 | 379/421 | - | 661 | 715 | 172 | 158 |
| 06TG293 | Day0 | Day42 | 210 | 188 | 153 | 153 | 157 | - | 283 | 291 | - | - | 685 | - | 174 | 151 |
| 06TG310 | Day0 | Day42 | 176 | 200 | 135 | - | - | 231 | 271 | - | - | 512 | 656 | 721 | 169 | 154 |
| 06TG321 | Day0 | Day28 | 179 | 148 | - | - | 188 | 196 | - | 183/300 | 448 | - | - | - | 168 | 155 |
| 02TG003 | Day0 | Day7 | 212 | - | - | - | 196 | 136 | - | - | 522 | 364 | 751 | - | 174 | 172 |
| 02TG019 | Day0 | Day21 | 197 | 219 | 144 | 149 | - | 136 | 280 | - | - | - | 708 | - | 175 | 154/184 |
| 02TG024 | Day0 | Day28 | 185 | 202 | 149 | 152 | 204 | 211 | 264 | 293 | 397 | 414 | - | 724 | 185 | 186 |
| 02TG033 | Day0 | Day28 | 212 | 205 | - | 157 | 169/210 | 207 | - | - | - | 394 | 669 | 717 | 180 | 212 |
| 02TG054 | Day0 | Day35 | 197 | 207 | 157 | - | - | 226 | - | - | - | 420 | 729 | - | 157 | 210 |
| 02TG076 | Day0 | Day35 | 158 | 185 | 163 | - | - | 215 | - | - | 422 | - | 772 | 735 | 158 | 211 |
| 02TG109 | Day0 | Day7 | 182 | 217 | - | 165 | - | 222 | - | - | - | 420 | 772 | - | 159 | 205 |
| 02TG113 | Day0 | Day42 | 212 | 163/200 | - | 165 | 207 | - | - | - | 406 | 409 | 713 | 536 | 176 | 177 |
| 02TG119 | Day0 | Day42 | 225 | 241 | - | 165 | 161/218 | - | - | - | 395/451 | 345 | - | 797 | 156 | 157 |
| 02TG132 | Day0 | Day35 | 200 | 230 | - | - | 203 | 197 | - | - | 423 | - | - | - | 190 | - |
| 02TG140 | Day0 | Day35 | 194 | 233 | - | 168 | 149 | 229 | - | - | 434/485 | 379 | - | - | 158 | 151/203 |
| 02TG173 | Day0 | Day28 | 191 | 219 | 136 | - | 191 | 147/258 | - | - | 392 | - | 425 | 733 | 161 | 159 |
| 02TG174 | Day0 | Day42 | - | 175/281 | - | - | - | 149 | - | 288 | - | - | - | - | - | 160 |
| 02TG180 | Day0 | Day28 | 280 | 188/289 | - | - | - | 207/252 | 281 | - | - | 503 | 743 | 665 | 187 | 156 |
| 02TG186 | Day0 | Day21 | 267 | 141 | 143 | 145 | 191 | - | - | 192 | 481 | - | 623/794 | 558/680 | - | 163 |
| 02TG196 | Day0 | Day42 | 136 | - | - | - | 176 | 179 | - | - | 422 | 468 | 616/673 | 559 | 179 | 170 |
| 02TG212 | Day0 | Day28 | 148/195 | 178 | 145 | - | 183 | - | 167/269 | 208,6 | 372 | - | 631/796 | 797 | 162 | 174 |
| 02TG216 | Day0 | Day28 | 171/222/274 | 206 | - | - | - | 167 | 272,5 | 184/266 | 368/533 | - | 789 | - | 174 | 155 |
| 02TG225 | Day0 | Day42 | 177/281 | - | 145 | "- | 186 | - | 267 | - | 402 | - | 606 | 613 | 153 | 152 |
| 02TG243 | Day0 | Day42 | - | - | - | - | 183 | 211 | 286 | - | 415 | - | 715 | 413 | 190 | 175 |
| 02TG271 | Day0 | Day42 | 165/286 | 176/294 | 145 | - | 231 | 237 | 280 | 277 | 469 | 468 | 654 | 701 | 151 | 169 |
| 02TG301 | Day0 | Day42 | 143/294 | - | 149 | 152 | 250 | - | 247 | - | - | 415 | 651 | 657 | 179 | 149 |
| 02TG325 | Day0 | Day28 | 167/288 | 169 | 154 | 154 | 241 | - | - | 255 | 387/436 | 483 | 740 | 616 | 171 | 158/198 |

| **ID** | **Day0** | **Dayx** | ***msp1*** | ***msp2*** | ***glurp*** | ***poly α*** | **Conclusion** | | **Concordance** |
| --- | --- | --- | --- | --- | --- | --- | --- | --- | --- |
|  |  |  |  |  |  |  | ***msp1/msp2/glurp*** | ***msp1/msp2/poly α*** |  |
| 06TG046 | Day0 | Day28 | Recrudescence | N/A | Recrudescence | Reinfection | **Recrudescence** | Reinfection | No |
| 06TG047 | Day0 | Day28 | Recrudescence | Reinfection | Reinfection | Reinfection | Reinfection | Reinfection | **Yes** |
| 06TG048 | Day0 | Day28 | Recrudescence | Reinfection | N/A | Reinfection | Reinfection | Reinfection | **Yes** |
| 06TG058 | Day0 | Day28 | Reinfection | Reinfection | N/A | Recrudescence | Reinfection | Reinfection | **Yes** |
| 06TG070 | Day0 | Day21 | Recrudescence | Recrudescence | Recrudescence | Reinfection | **Recrudescence** | Reinfection | No |
| 06TG114 | Day0 | Day35 | N/A | N/A | N/A | N/A | *N/A* | *N/A* | **Yes** |
| 06TG153 | Day0 | Day21 | Recrudescence | Recrudescence | Reinfection | Recrudescence | Reinfection | **Recrudescence** | No |
| 06TG159 | Day0 | Day35 | Recrudescence | Recrudescence | Reinfection | Reinfection | Reinfection | Reinfection | **Yes** |
| 06TG184 | Day0 | Day42 | Reinfection | N/A | Reinfection | Recrudescence | Reinfection | Reinfection | **Yes** |
| 06TG186 | Day0 | Day35 | Recrudescence | N/A | Recrudescence | Recrudescence | **Recrudescence** | **Recrudescence** | **Yes** |
| 06TG189 | Day0 | Day28 | N/A | Reinfection | Recrudescence | Reinfection | Reinfection | Reinfection | **Yes** |
| 06TG195 | Day0 | Day21 | Recrudescence | N/A | N/A | N/A | *N/A* | *N/A* | **Yes** |
| 06TG212 | Day0 | Day28 | Reinfection | Reinfection | Reinfection | Reinfection | Reinfection | Reinfection | **Yes** |
| 06TG287 | Day0 | Day21 | Recrudescence | Recrudescence | Reinfection | Reinfection | Reinfection | Reinfection | **Yes** |
| 06TG293 | Day0 | Day42 | Recrudescence | Recrudescence | N/A | Reinfection | **Recrudescence** | Reinfection | No |
| 06TG310 | Day0 | Day42 | Reinfection | N/A | Reinfection | Reinfection | Reinfection | Reinfection | **Yes** |
| 06TG321 | Day0 | Day28 | Recrudescence | Reinfection | N/A | Reinfection | Reinfection | Reinfection | **Yes** |
| 02TG003 | Day0 | Day7 | Reinfection | Reinfection | N/A | Recrudescence | Reinfection | Reinfection | **Yes** |
| 02TG019 | Day0 | Day21 | Recrudescence | N/A | N/A | Recrudescence | *N/A* | **Recrudescence** | No |
| 02TG024 | Day0 | Day28 | Recrudescence | N/A | N/A | Recrudescence | *N/A* | **Recrudescence** | No |
| 02TG033 | Day0 | Day28 | Recrudescence | N/A | Recrudescence | Reinfection | **Recrudescence** | Reinfection | No |
| 02TG054 | Day0 | Day35 | Recrudescence | N/A | N/A | Reinfection | *N/A* | Reinfection | No |
| 02TG076 | Day0 | Day35 | Reinfection | N/A | Recrudescence | Reinfection | Reinfection | Reinfection | **Yes** |
| 02TG109 | Day0 | Day7 | Reinfection | N/A | N/A | Reinfection | *N/A* | Reinfection | No |
| 02TG113 | Day0 | Day42 | Recrudescence | Recrudescence | Reinfection | Recrudescence | Reinfection | **Recrudescence** | No |
| 02TG119 | Day0 | Day42 | Recrudescence | Recrudescence | N/A | Recrudescence | **Recrudescence** | **Recrudescence** | **Yes** |
| 02TG132 | Day0 | Day35 | Recrudescence | N/A | N/A | N/A | *N/A* | *N/A* | **Yes** |
| 02TG140 | Day0 | Day35 | Reinfection | Reinfection | N/A | Recrudescence | Reinfection | Reinfection | **Yes** |
| 02TG173 | Day0 | Day28 | Reinfection | N/A | Reinfection | Recrudescence | Reinfection | Reinfection | **Yes** |
| 02TG174 | Day0 | Day42 | N/A | N/A | N/A | N/A | *N/A* | *N/A* | **Yes** |
| 02TG180 | Day0 | Day28 | Recrudescence | Reinfection | Reinfection | Reinfection | Reinfection | Reinfection | **Yes** |
| 02TG186 | Day0 | Day21 | Recrudescence | Reinfection | Reinfection | N/A | Reinfection | Reinfection | **Yes** |
| 02TG196 | Day0 | Day42 | Recrudescence | Reinfection | Reinfection | Recrudescence | Reinfection | Reinfection | **Yes** |
| 02TG212 | Day0 | Day28 | Recrudescence | Reinfection | Recrudescence | Reinfection | Reinfection | Reinfection | **Yes** |
| 02TG216 | Day0 | Day28 | Recrudescence | Recrudescence | N/A | Reinfection | **Recrudescence** | Reinfection | No |
| 02TG225 | Day0 | Day42 | N/A | N/A | Recrudescence | Recrudescence | *N/A* | *N/A* | **Yes** |
| 02TG243 | Day0 | Day42 | Reinfection | N/A | Reinfection | Reinfection | Reinfection | Reinfection | **Yes** |
| 02TG271 | Day0 | Day42 | Recrudescence | Recrudescence | Recrudescence | Reinfection | **Recrudescence** | Reinfection | No |
| 02TG301 | Day0 | Day42 | Recrudescence | Reinfection | Recrudescence | Reinfection | Reinfection | Reinfection | **Yes** |
| 02TG325 | Day0 | Day28 | Recrudescence | Reinfection | Reinfection | Reinfection | Reinfection | Reinfection | **Yes** |
